# From Fish Scale Gelatin to Tyrosinase Inhibitor: A Novel Peptides Screening Approach Application

**DOI:** 10.3389/fnut.2022.853442

**Published:** 2022-03-18

**Authors:** Zi-Zi Hu, Xiao-Mei Sha, Lu Zhang, Min-Jun Zha, Zong-Cai Tu

**Affiliations:** ^1^National R&D Center for Freshwater Fish Processing, College of Chemistry and Chemical Engineering & College of Life Science, Jiangxi Normal University, Nanchang, China; ^2^State Key Laboratory of Food Science and Technology, Nanchang University, Nanchang, China

**Keywords:** grass carp scale gelatin, tyrosinase inhibitory peptides, bioaffinity ultrafiltration, LC-Orbitrap-MS/MS, whitening

## Abstract

Bioaffinity ultrafiltration combined with LC-Orbitrap-MS/MS was applied for the first time to achieve rapid screening and identification of tyrosinase inhibitory peptides (TYIPs) from grass carp scale gelatin hydrolysates. The binding mode of TYIPs with tyrosinase was investigated by molecular docking technology. The whitening effect of TYIPs was further studied by evaluating the tyrosinase activity and melanin content in mouse B16F10 cells. Four new TYIPs were screened from hydrolysates, among which DLGFLARGF showed the strongest tyrosinase inhibition with an IC_50_ value of 3.09 mM. Molecular docking showed that hydrogen bonds were the main driving force in the interaction between the peptide DLGFLARGF and tyrosinase. The addition of DLGFLARGF significantly inhibited the tyrosinase activity and melanin production of B16F10 melanoma cells. These results suggest that DLGFLARGF is a promising skin whitening agent for the treatment of potential pigment-related diseases.

## Introduction

Gelatin has a rich sources and is widely used in the food and medicine industries (1–5). The hydrolysis product of gelatin is collagen peptide, which is a mixture of peptides. After separation and purification, specific bioactive peptides could be obtained that have a wide range of significant functional activities. At present, the functional activities of collagen peptides such as antioxidant activity (6), angiotensin-converting enzyme inhibition activity (7), anti-tumor activity (8), and antibacterial activity (9) have been studied. Recent studies have shown that many active peptides contain some hydrophobic amino acids, uncharged polar amino acids, aromatic amino acids, etc., which have the ability to inhibit the production of melanin (10, 11).

The production of melanin is regulated by enzymes such as tyrosinase, tyrosinase-binding protein-1 (dopa pigment isomerase), and tyrosinase-binding protein-2 (melanin precursor oxidase) (12). Tyrosinase (EC1.14.18.1) is a multifunctional enzyme with three histidine (His) residues and two Cu ions in the active center, which is widely found in fungi, plants and animals. Tyrosinase oxidizes tyrosine to produce dopamine and then continues to oxidize dopamine to produce dopamine quinone. Dopamine quinone is a very active molecule that can generate a polymer complex or brownish pigment when it reacts with amino acids or proteins, finally producing melanin (13). Substances with tyrosinase inhibition activity are commonly found in whitening foods. At present, tyrosinase inhibitors widely used in foods have been reported, including arbutin, kojic acid, oxidative resveratrol, etc. (14). However, studies have shown that these substances show limiting effects and certain side effects, including the lack of good permeability, high toxicity and poor stability (15). Scientists have recently become interested in the tyrosinase inhibitory activity of extracts from natural sources, due to the benefits of mild functional activity, simple absorption, and excellent skin compatibility (16–18). Active polypeptide is composed of 2–20 amino acids, which has the above advantages that could provide the possibility for its application in foods (19).

The conventional procedures for screening active peptides from complex protein hydrolysates include the preparation of hydrolysates, bioassay-guided separation, purification and peptide sequence identification. In general, traditional methods require multi-step extraction and separation with organic solvents, resulting in low efficiency, serious environmental pollution, time-consuming and laborious (20, 21). The combination of bioaffinity ultrafiltration and liquid chromatography-mass spectrometry based on the interaction between small molecule ligands and enzyme active sites is an effective approach for a powerful approach for identifying biologically active compounds from complex mixtures (22). It has been widely used to screen and identify a variety of biologically active compounds from natural extracts and traditional Chinese medicine (23). Qin et al. (24) established a bioaffinity ultrafiltration-high performance liquid chromatography-electrospray ionization-time of flight-mass spectrometry (BAUF-HPLC-ESI-TOF/MS) method to identify potential new bioactive substances. This method has been demonstrated to be a quick way to obtain high-purity, high-activity bioactive substances. However, there is no report about screening tyrosinase inhibitor peptides by this method.

In this paper, a rapid screening and identification method for the tyrosinase inhibitory peptides (TYIPs) in the hydrolysis of fish scale (by-products during freshwater fish production) gelatin was established for the first time based on ultrafiltration and Nano-LC-Orbitrap-MS/MS. Then, molecular docking was used to determine the interaction of the identified peptide with tyrosinase in order to investigate the biological activity and mechanism. Finally, the effect of peptides on B16F10's ability to produce melanin was further studied.

## Materials and Methods

### Materials

Grass carp scale gelatin was obtained from the laboratory. Alcalase (≥200,000 units/g) was purchased from Solarbio Chemical Co. (Shanghai, China). Mushroom tyrosinase (6,680 U/mg) and 3,4-dihydroxyphenylalanine (L-DOPA) were purchased from Sigma-Aldrich (St. Louis, MO, USA). The murine melanoma B16F10 cells (CL0039) were acquired from Fenghui Biological Technology Co., Ltd. (Hunan, China). All the other reagents were analytical grade.

### Preparation of TYIPs From Fish Scale Gelatin

The grass carp scale gelatin was prepared according to the method of Sha et al. (25). According to previous experiments, the optimal enzymatic hydrolysis conditions for preparing TYIPs were: substrate concentration of 125 mg/ml, alcalase dosage of 1%, pH 9.0, enzymatic hydrolysis temperature of 60°C, and enzymatic hydrolysis time of 2 h.

### Analysis of the Tyrosinase Inhibitory Activity

The determination of the tyrosinase inhibitory activity was made according to Uysal et al. (26). Sample solutions (50 μl) with the appropriate concentration were reacted with 50 μl of 200 mM pH 6.8 phosphate buffer saline (PBS) solution and 50 μl of tyrosinase at room temperature for 15 mins. Then, 50 μl of 2.5 mM L-DOPA was added, and allowed to react at 30°C for 10 min. The absorbance (As) at 475 nm was finally measured with a microplate reader (Synergy H1, BioTek Co., Ltd., USA). Taking the reaction system without enzyme and sample as a blank. Kojic acid (2 mg/ml) used as the positive control, and the rate of tyrosinase inhibition could be calculated using the following equation:

(1) $$\begin{array}{r} Tyrosinase\;inhibition\;rate/\%=\frac{\left(A_{c}-A_{b}\right)-\left(A_{s}-A_{b}\right)}{\left(A_{c}-A_{b}\right)}\times 100\% \end{array}$$

where, As is the absorbance of the sample after reaction; Ac is the absorbance of the reaction system with distilled water instead of the sample; Ab is the absorbance of the reaction system with distilled water instead of tyrosinase.

### Analysis of the Amino Acid Composition

The amino acid composition was determined based on the report of Chen et al. (27) with appropriate modifications. The samples were hydrolyzed with 6 M HCl at 110°C for 24 h prior to composition analysis with a High-Speed Amino Acid Analyzer Model L-8900 (Hitachi Co., Japan).

### Analysis of Molecular Weight Distribution

The molecular weight (MW) distribution was determined using an Agilent 1260 Infinity HPLC system (Agilent Technologies, Inc., Santa Clara, CA, USA) equipped with a waters XBridge Protein BEH 125Å SEC (3.5 μm, 7.8 × 300 mm) (28). Hydrolysates were eluted with water (0.1% FA) and acetonitrile (60:40, V/V) at a flow rate of 0.4 ml/min. The injection volume was 10 μl. The detection wavelength was 220 nm (28). The MW of peptides was calculated based on the calibration curve constructed with cytochrome C (12,384 Da), aprotinin (6,511.51 Da), bacitracin (1,422.69 Da), L-oxidized glutathione (612.63 Da) and hydroxyproline (131.13 Da).

### Rapid Screening of TYIPs

#### Bioaffinity Ultrafiltration

The screening method was slightly modified based on the previous report (22). The inhibitory activity of tyrosinase was evaluated at 1–6 mg/ml to obtain the best binding concentration.

4 ml of hydrolysis product (5 mg/ml) and 2 ml of mushroom tyrosinase (10 U/ml) were incubated for 1 h at 37°C. Inactivated mushroom tyrosinase (heated in a boiling water bath for 10 mins) was also prepared as a blank group. The detailed method is shown in **Figure 2A**. Filtrates, including peptides binding active tyrosinase (PAT) and peptides binding inactive tyrosinase (PIT), were collected and freeze-dried for tyrosinase inhibition evaluation and peptide identification.

#### Peptide Identification

The amino acid sequence of the components obtained in 2.6.1 was determined by Nano-LC-ESI-Q-Orbitrap-MS/MS. Peptides were separated on an AcclaimR PepMap RSLC (50 μm × 150 mm, C18, 2 μm, 100 Å) column at a flow rate of 220 nl/min. Mobile phase A and B was consisted of 0.1% formic acid aqueous solution and 0.1% acetonitrile solution, respectively. Gradient elution conditions were as follows: 0–2 min, 4–12% B; 2–25 min, 12–22% B; 25–32 min, 22–32% B; 32–37 min, 32–75% B; 37–40 min, 75% B (isoelution). The positive ion scanning mode was adopted, and the mass spectrometry data were collected using Xcalibur 2.2 SP1 software, with a mass range of 250–1,250 m/z and a resolution of 70,000. The top 20 peptides were selected for fragmentation according to the signal strength of the first mass spectrometry, and the fragmentation mode was HCD with an energy of 27%. The parent ion map was analyzed by Xcalibur software and De Novo was sequenced using PEAKS Studio 7.0 software to obtain the amino acid sequence of peptides. The peptides identified in this paper met the requirements of false discovery rate (FDR) ≤ 5% and average local confidence score (ALC) ≥ 95%.

### Molecular Docking and Peptides Synthesis

The X-ray crystal structure of Agaricus bisporus tyrosinase (2Y9W) was downloaded from the Protein Data Bank (https://www.rcsb.org/structure/2Y9W) (29), and three-dimensional (3D) structure of TYIPs segment were obtained by ChemBio 3D Ultra 14.0. Based on the 3D crystal structure of tyrosinase, the computer-aided technology was used to analyze the action mode and site of peptide segment with tyrosinase, and to clarify the action mechanism. The specific docking process was as follows: first, the AutoDock tool is used to remove water molecules from tyrosinase and add Gasteiger charges and hydrogen atoms to tyrosinase molecules. Then, the AutoDock tool was used again to dock ligand small molecules (peptides) with tyrosinase. The docking process and calculation were carried out according to default parameters. The coordinates of the docking center of the peptide with tyrosinase were (64, 70, 116). Results were obtained based on the lowest free energy.

### Cell Culture

B16F10 cells were kept in Dulbecco's modified eagle medium supplemented with 10% heat-inactivated fetal bovine serum, and cells were cultured at 37°C in an atmosphere with 5% CO_2_. These cells were then used for cell viability, tyrosinase inhibition and melanin content determination.

### Determination of the Cell Viabilities of B16F10 Melanoma Cells

The previously described cell counting kit-8 (CCK-8) assay was used to assess cell viability (30). The cells were incubated in a 96-well plate with 5 × 10^4^ cells per well in a 5% humid CO_2_ atmosphere at 37°C for 24 h. DLGFLARGF was added to the cells at concentrations of 0, 0.1, 0.2, 0.4, 0.8 and 1.6 mg/ml, while 0.75 mg/ml of kojic acid was used as a positive control. The cells were re-incubated for 24 h under the same conditions. The cells were then treated with the CCK-8 reagent and incubated at 37°C for 1 h. Cell viability is calculated by reading the absorbance value at 450 nm. The calculation formula is shown below.

(2) $$\begin{array}{r} Cell\;viability/\%=\frac{\left(A_{s}-A_{b}\right)}{\left(A_{c}-A_{b}\right)}\times 100\% \end{array}$$

where, As is the absorbance of experimental wells (medium containing cells, CCK-8, sample to be tested); Ac is the absorbance of control well (medium containing cells, CCK-8, no sample to be tested); Ab is the absorbance of blank wells (medium without cells and samples to be tested, CCK-8).

### Determination of Tyrosinase Activity in B16F10

The tyrosinase inhibition was determined according to the method of Ullah et al. (31). B16F10 cells were seeded into 96-well plates at a rate of 5 × 10^4^ cells per well and incubated at 37°C in a humid atmosphere of 5% CO_2_ for 24 h. The cells were then treated with kojic acid (0.75 mg/ml) and DLGFLARGF (0, 0.1, 0.2, 0.4, 0.8 and 1.6 mg/ml), and re-incubated for 24 h under the same conditions. The cells were then washed with PBS buffer, lysed with lysate buffer [100 μl containing 50 mM PBS, 0.1 mM phenylmethanesulfonyl fluoride (5 μl) and Triton X-100 (5 μl)], and frozen at −80°C for 30 min. The cell lysate was then centrifuged at 12,000 rpm at 4°C for 30 min and transferred to a 96-well plate with a total volume of 100 μl (80 μl lysate supernatant and 20 μl 10 mM L-DOPA) and incubated for 30 min at 37°C. The absorbance was measured at 450 nm using an enzyme-linked immunosorbent assay (ELISA) reader.

### Determination of Melanin Content in B16F10

After cultivated according to the instructions above, the cells were digested with 0.25% trypsin when reached to 80–90% confluence to make a single cell suspension. Fresh culture medium was used to adjust the cell concentration to 1 × 10^6^ cells/ml, 2 ml of which was inoculated in a 6-well culture plate for incubation overnight. When the cells adhered, the supernatant was discarded and the cells were rinsed with PBS once. 2 ml fresh culture medium was added again for incubation under CO_2_ for 48 h. The supernatant was discarded after the incubation, and the adhered cells were rinsed with PBS and then dispersed with trypsin. The dispersed cells were collected and centrifuged at 1,500 r/min for 10 min. The precipitation was added with 1 ml NaOH (1 mol/L) containing 10% dimethyl sulfoxide for 1 h water bath at 80°C before being transferred to a 96-well culture plate. Measured the optical density value of each well at 405 nm with a microplate reader, and calculated the melanin content (32).

(3) $$\begin{array}{r} Melanin\;content/\%=\frac{\left(A_{s}-A_{b}\right)}{\left(A_{c}-A_{b}\right)}\times 100\% \end{array}$$

where, As is the absorbance of experimental wells; Ac is the absorbance of control well; Ab is the absorbance of blank wells.

### Statistical Analysis

In each analysis, three parallel tests were performed. The results were presented in the form of mean ± standard deviation (SD). SPSS Statistics 20 software (IBM, Armonk, NY, USA) was used to perform one-way analysis of variance (ANOVA, *P* < 0.05) and Duncan's multiple range test to analyze the differences between samples.

## Results and Discussion

### Amino Acid Composition and Molecular Weight Distribution of Hydrolysates

The amino acid composition of hydrolysates was shown in Table 1 and the total amount of amino acids was 67.12 g in 100 g hydrolysates. Many amino acid residues in collagen peptides are associated to tyrosinase inhibitory activity, according to the structure-activity relationship investigation between the peptide chain and melanin production inhibition (10, 33, 34). Val (V), Ala (A), Leu (L) and Ile (I) are four aliphatic hydrophobic amino acids that can directly interact with enzymes to inhibit the formation of dopaquinone, hence inhibiting melanin production, and have an additive effect. As shown in Table 1, the total amount of these four amino acids was 11.04 g/100 g hydrolysates, accounting for 16.45% of the total amino acid content. Arg (R) residues enhance cell penetration and facilitate the interaction between peptides and tyrosinase; Phe (F) is similar in structure to Tyr (Y) (the natural substrate of tyrosinase), which facilitates the binding of peptides and enzymes (10). The contents of these two amino acids were 6.74 g/100 g and 1.53 g/100 g in hydrolysates, respectively. Cys (C), Ser (S) and Thr (T) can form a complex with the enzymatic reaction product (dopaquinone) to prevent the conversion of dopaquinone into melanin, instead of inhibiting enzyme activity. The total amount of these three amino acids was 4.54 g/100 g in hydrolysates. Asp (D) and Glu (E) are negatively charged amino acid residues, which are not conducive to binding to tyrosinase. The total amount of these two amino acids was 12.40 g/100 g hydrolysates, accounting for 18.47% of the total amino acid. From what has been discussed above, the content of amino acids beneficial to inhibiting melanin was up to 35.44%. These results suggested that hydrolysates may have strong tyrosinase inhibitory activity.

**Table 1:** Amino acid composition of fish scale gelatin hydrolysate.

**Amino acid**	**Content (g per 100 g)**	**Amino acid**	**Content (g per 100 g)**
Asp	4.58 ± 0.16	Ile^*^	1.03 ± 0.02
Thr	2.00 ± 0.05	Leu^*^	2.60 ± 0.08
Ser	2.49 ± 0.05	Tyr	0.41 ± 0.05
Glu	7.82 ± 0.16	Phe^*^	1.53 ± 0.03
Gly	16.71 ± 0.03	Lys	2.83 ± 0.03
Ala^*^	5.58 ± 0.04	His	0.26 ± 0.01
Cys	0.05 ± 0.01	Arg	6.74 ± 0.01
Val^*^	1.83 ± 0.04	Pro^*^	9.43 ± 0.07
Met^*^	1.24 ± 0.07		

* *Hydrophobic amino acid*.

**Figure 1:**
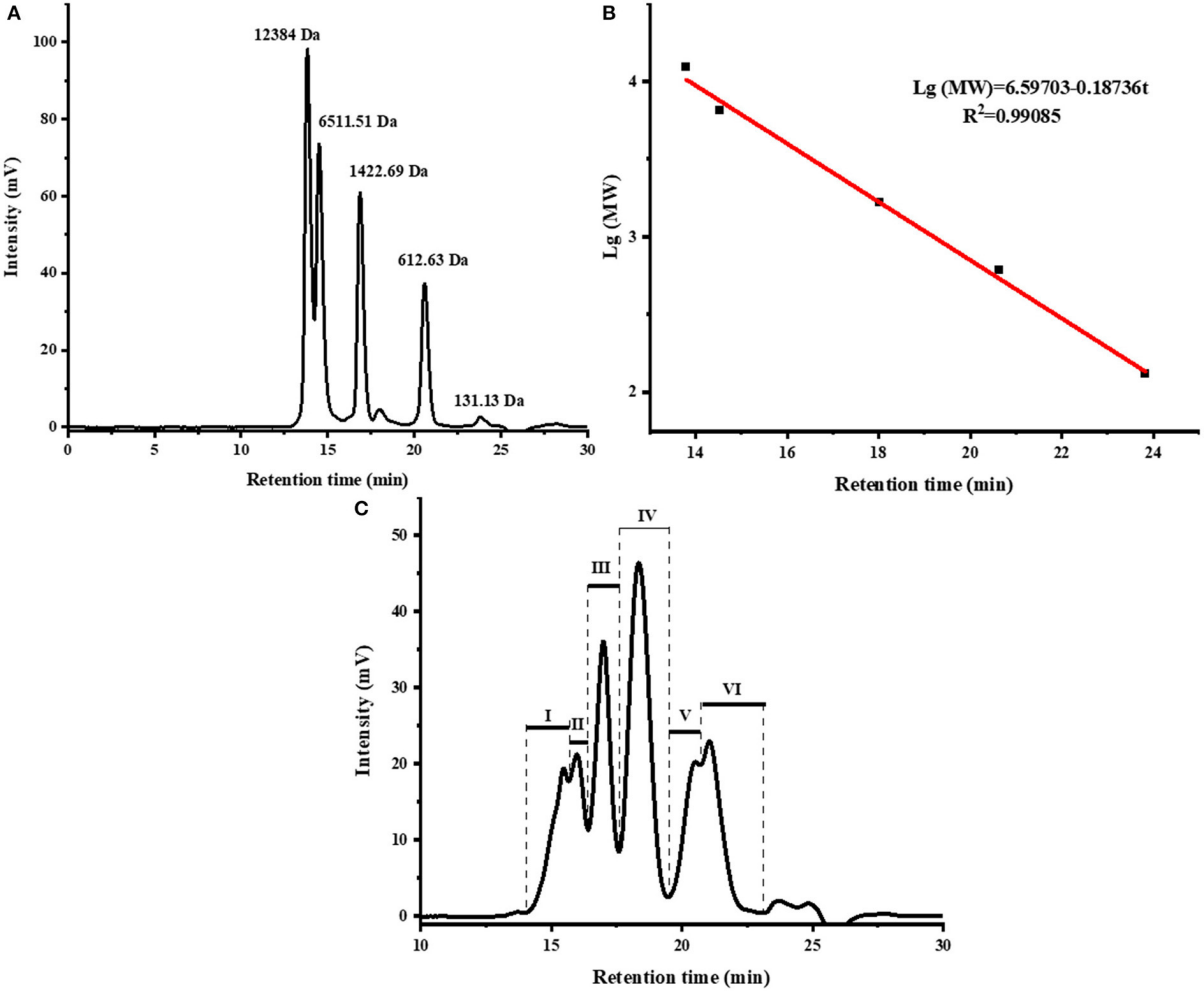
The size exclusion chromatogram of standards **(A)**, molecular weight distribution curve of standards **(B)**, and the size exclusion chromatogram of fish scale gelatin hydrolysates, I, II, III, IV, V, VI represent the different components obtained **(C)**.

The MW distribution reflects the hydrolysis degree of fish scale gelatin (35). Peptides with different MW have different tyrosinase inhibition abilities (36). As shown in Figure 1 and Table 2, there were mainly six peaks (I-VI), indicating that the hydrolysate contained six components, and the MW distribution of components I, II, III, IV, V and VI were 9,217–4,642 Da, 4,642–3,358 Da, 3,358–1,992 Da, 1,992–904 Da, 904–503 Da and 503–180 Da, respectively. The MW of the hydrolysates were mainly concentrated in components III, IV, V and VI, whose relative contents were 17.05, 29.65, 10.51, and 16.12%, accounting for more than 73% of the total contents. In addition, the MW of hydrolysates was mainly concentrated below 1,992 Da, accounting for 56.28% of proteolytic products. Alcalase is a non-specific protease that can cleave many sites in a protein at random and generate a higher number of low MW peptides (37), which are attractive for higher biofunctional activity (38).

**Table 2:** Molecular weight distribution of fish scale gelatin hydrolysate.

**Component**	**Molecular weight range (Da)**	**Retention time (min)**	**Relative content (%)**	**Peak molecular weight (Da)**
I	9,217–4,642	14.05–15.64	9.75	4,974
II	4,642–3,358	15.64–16.39	8.80	4,044
III	3,358–1,992	16.39–17.60	17.05	2,560
IV	1,992–904	17.60–19.43	29.65	1,441
V	904–503	19.43–20.67	10.51	568
VI	503–180	20.67–23.17	16.12	452

### Enrichment and Identification of Potential TYIPs

**Figure 2:**
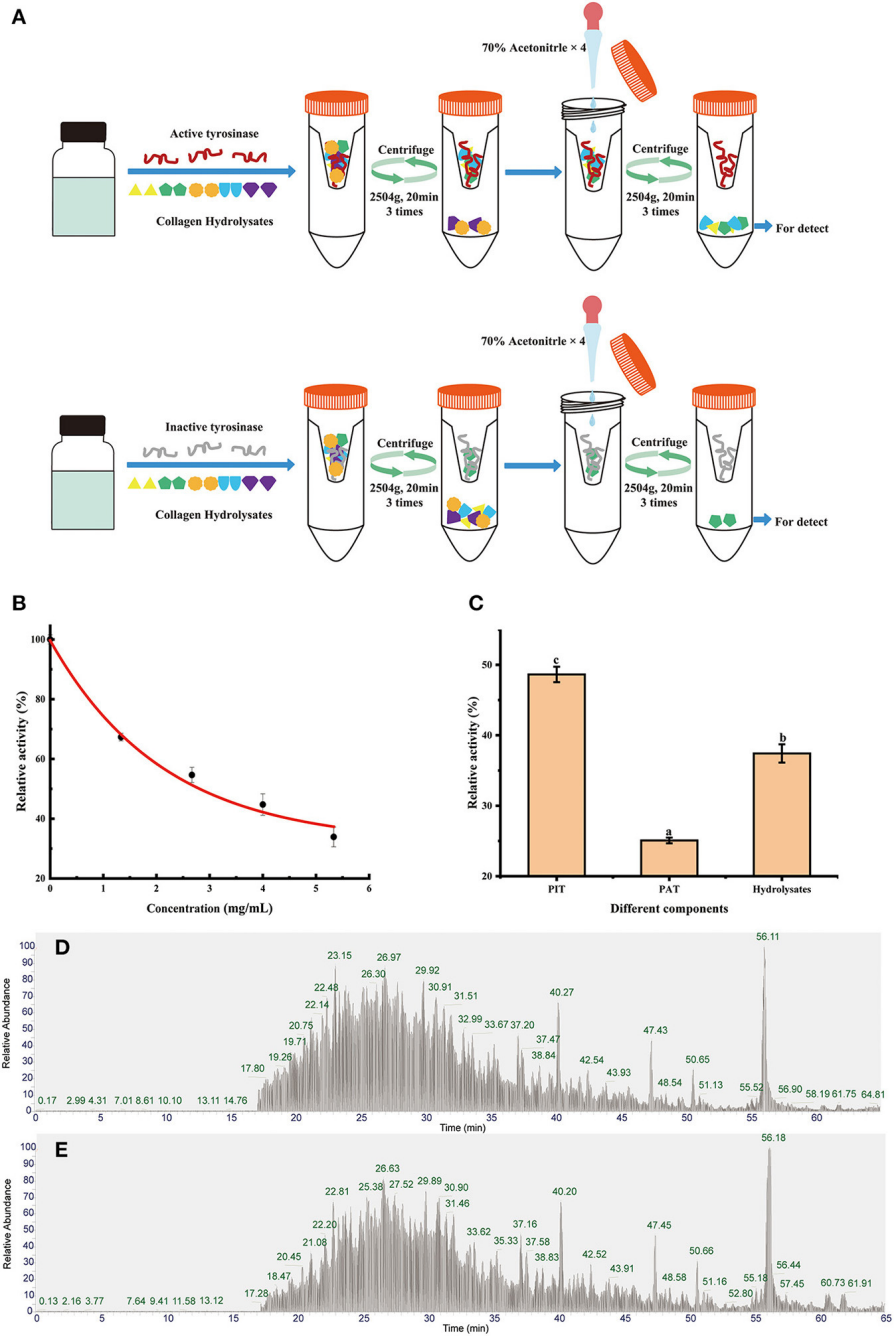
Diagrams of biological affinity ultrafiltration—liquid mass spectrometry screening methods **(A)**, the tyrosinase activity of hydrolysates **(B)**, ultrafiltration fractions **(C)**, the total ion chromatography (TIC) of PAT **(D)**, and PIT **(E)**. Different lowercase letters represent significant differences between different groups (*p* < 0.05).

Appropriate concentration and ratio are required in order to achieve a saturated state of binding. Figure 2B shows the tyrosinase activity residual rate of hydrolysates with different concentrations. Significant dose-dependence was observed in the concentration range of 0–5.33 mg/ml, which indicated that most of the active sites of tyrosinase can be bound by peptides. However, the inhibitory effect per milligram of inhibitor decreased as the hydrolysates concentration increased. In order to retain an ideal tyrosinase inhibitory activity, the concentration of hydrolysates was set at 5 mg/ml with tyrosinase inhibitory activity rate at 61.7% for screening.

After bioaffinity ultrafiltration, tyrosinase inhibitions of PAT and PIT were evaluated. The results are shown in Figure 2C. The inhibition rate of tyrosinase on PAT was 74.92%, while the corresponding inhibition rates of PIT and hydrolysates were 51.37 and 62.58%, respectively. This suggests that certain peptides in the hydrolysates can specifically bind to the active site of tyrosinase and can be enriched effectively by bioaffinity ultrafiltration, resulting in a higher inhibition of PAT than PIT and hydrolysates.

**Figure 3:**
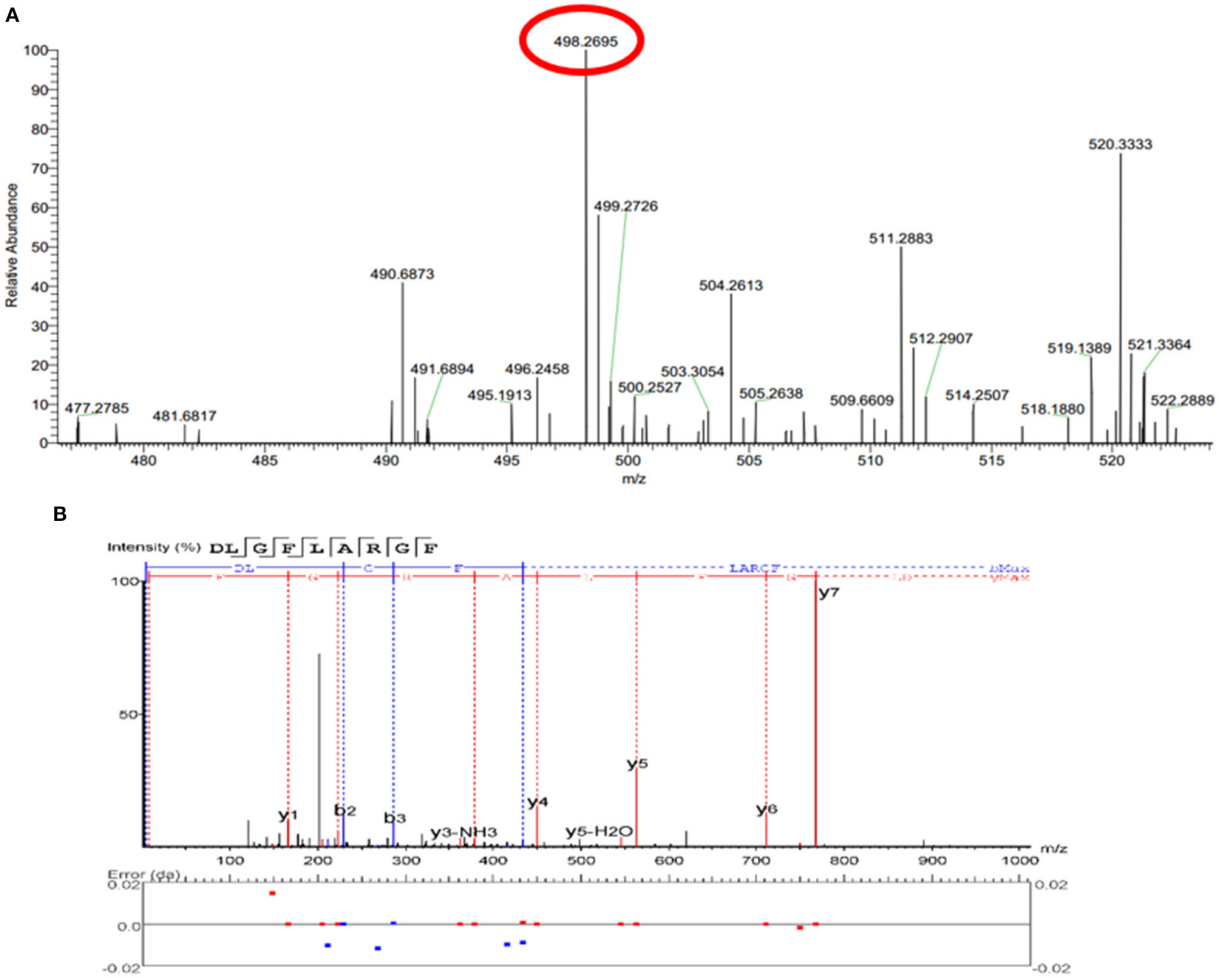
The full MS scan spectrum **(A)** and the full MS/MS spectrum **(B)** of DLGFLARGF.

The peptide composition in PAT and PIT was identified by Nano-LC-Q-Orbitrap-MS/MS to screen potential TYIPs. The total ion chromatograms were shown in Figures 2D,E. By matching the b and y series ions detected in the MS/MS spectrum with that recorded in database (uniprot-taxonomy Anabantaria 201912), the exact amino acid sequence of each peptide can be drawn. For example, peptide 9 (Figure 3A) has MS ion 498.2695^2+^, b series of b2 (229.1183^2+^), b3 (286.1397^2+^) and b4 (433.2082^2+^), y1 (166.0863^2+^), y2 (223.1077^2+^), y3 (379.2088^2+^), y4 (450.2459^2+^), y5 (563.3300^2+^), y6 (710.3984^2+^) and y7 (767.4199^2+^), and the sequence was identified as DLGFLARGF (Figure 3B). By this method, 52 of the peptides identified in PAT were not found in PIT. Inhibitors can bind specifically to the active site of tyrosinase and be released by acetonitrile (39), which confirmed the high selectivity of bioaffinity ultrafiltration. Therefore, these specific peptides in PAT are considered as potential TYIPs.

### Rapid Screening and Tyrosinase Inhibitory Activity Verification

**Figure 4:**
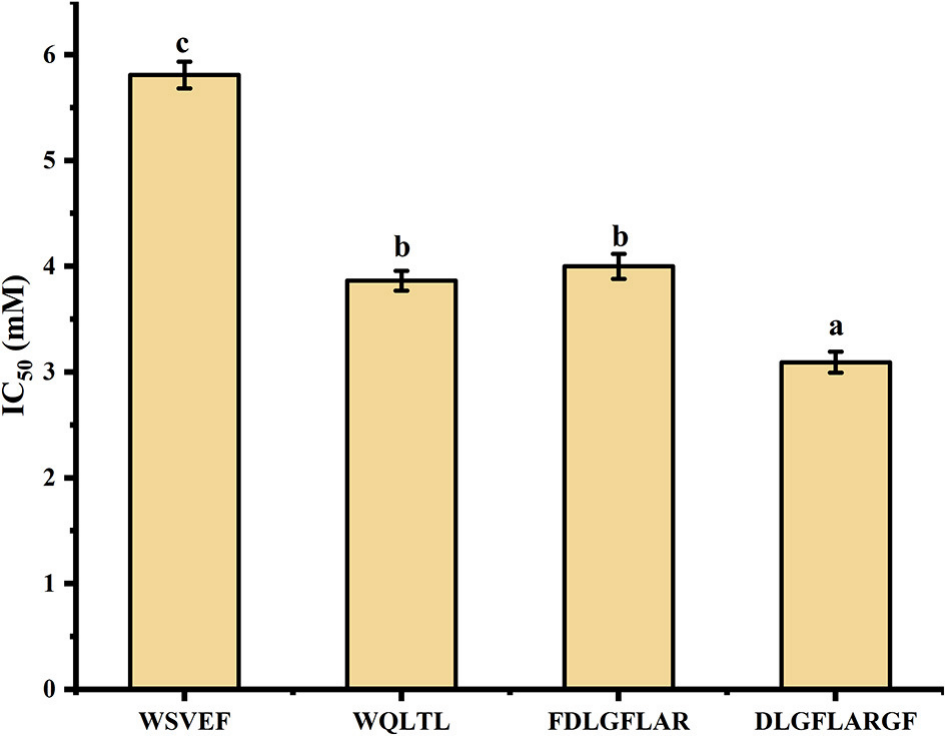
Tyrosinase inhibitory activity of synthetic peptides. Different lowercase letters represent significant differences between different groups (*p* < 0.05).

Studies have shown that peptides with lower MW tend to have higher functional activity (38). Therefore, peptides with MW ≤ 1,000 Da were selected from the 52 peptides obtained, and 16 peptides (shown in Table 3) were obtained for subsequent analysis. Section Amino Acid Composition and Molecular Weight Distribution of Hydrolysates introduced some amino acids that contribute to tyrosinase inhibition, such as Val, Ala, Leu, Ile, Arg, Phe, Cys, Ser, and Thr. According to the screening method from previous study (10), 16 peptides in Table 3 were counted for their tyrosinase inhibitory contribution ratio of amino acids, and 4 peptides (marked ^*^ in Table 3) with a contribution ratio of amino acids greater than or equal to 0.6 were obtained, they were discovered to be novel peptides by searching the data on the website http://www.uwm.edu.pl/biochemia/index.php/en/biopep. Therefore, the four peptides were used for subsequent synthesis to verify the tyrosinase inhibitory activity *in vitro*, the purity of the synthetic peptides was more than 95%. The tyrosinase inhibition ability of the synthetic peptide was shown in Figure 4. The peptide DLGFLARGF showed the strongest tyrosinase inhibition ability, with an IC_50_ value of 3.09 mM. The IC_50_ of peptides WQLTL, WSEVF and FDLGFLAR were 3.86, 5.81, and 4.00 mM, respectively, and they all showed good tyrosinase inhibitory activity. Similar peptides from natural sources have also been found in other studies, such as Phe-Pro-Tyr (FPY) from defatted walnut (*Juglans regia* L.) meal hydrolysate with an IC_50_ value of 3.22 ± 0.22 mM (40), and RHAKF from Chinese quince seed protein hydrolysate with IC_50_ value of 1.15 mg/ml (41). In order to further explore the reasons for the difference in tyrosinase inhibition of these four peptides, molecular docking analysis was performed.

**Table 3:** Identification of peptides unique to PAT by LC–ESI–Q–Orbitrap–MS/MS.

**No**.	**Peptide**	**RT (min)**	**Length**	**ALC (%)**	**m/z**	**Mass**	**Local confidence (%)**
1	PGPVGVKL	27.98	8	99	383.7446	765.4749	99 100 100 100 100 100 100 100
2	LDALNENK	20.2	8	99	458.7404	915.4661	100 100 100 99 98 99 98 100
3	VPGPM	25.62	5	98	500.2538	499.2465	99 99 100 99 98
4	GPVGSF	29.02	6	98	563.2829	562.2751	97 98 99 99 100 100
5	TGPLGL	34.4	6	98	557.3301	556.322	97 98 99 99 99 100
6	FDLGFLAR*	42.62	8	98	469.7591	937.5021	99 99 99 98 98 97 97 99
7	FSGM	24.61	4	97	441.1804	440.1729	96 96 100 99
8	WSVEF*	45.45	5	97	667.3090	666.3013	93 95 100 100 100
9	GEPGLLGM	42.87	8	97	773.3893	772.3789	89 95 97 98 99 100 99 100
10	GPPGLGQR	20.59	8	96	391.2201	780.4242	99 99 100 98 97 94 90 95
11	WQLTL*	47.88	5	96	660.3729	659.3643	97 94 99 96 95
12	EAPDPF	50.31	6	95	675.3026	674.2911	96 94 97 98 92 96
13	LVGPAGPTQR	21.96	10	95	498.2855	994.5560	100 100 100 100 99 99 91 86 85 93
14	DAPGLLRGF	35.7	9	95	473.2618	944.5079	79 88 100 100 100 99 97 95 99
15	DLGFLARGF*	45.92	9	95	498.2695	994.5236	81 82 99 100 100 99 98 99 99
16	GFTGM	28.03	5	95	512.2175	511.2101	90 92 98 99 97

The above refers to the molecular mass less than or equal to 1,000 Da. The proportion of amino acids that are marked with * means that the contribution of tyrosinase inhibition is greater than or equal to 0.6.

### Molecular Docking

**Figure 5:**
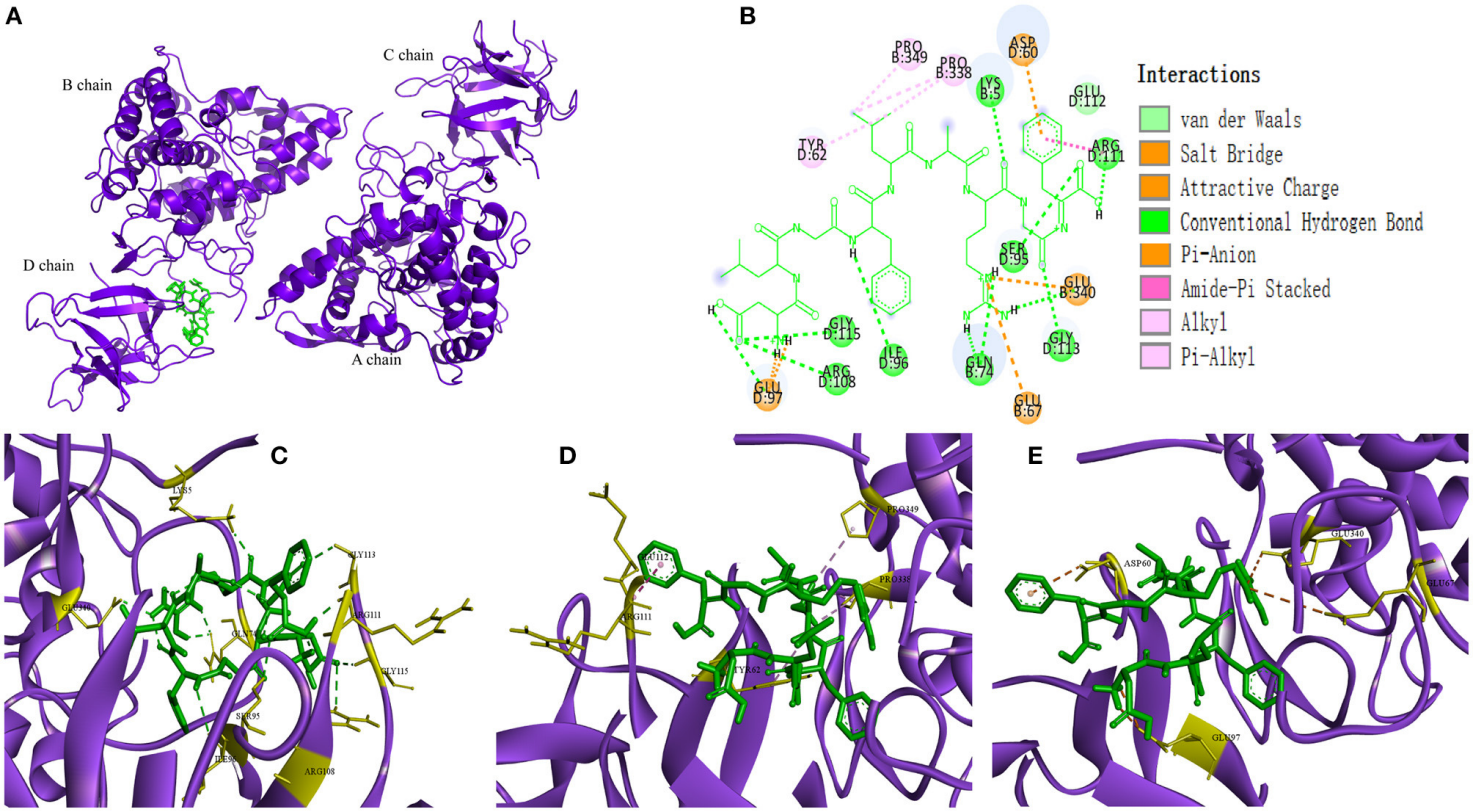
Predicted binding mode of DLGFLARGF and tyrosinase **(A,B)**. Hydrogen bond formed by DLGFLARGF and amino acid residues of tyrosinase **(C)**. Hydrophobic interaction between DLGFLARGF and amino acid residues of tyrosinase **(D)**. Electrostatic interaction between DLGFLARGF and amino acid residues of tyrosinase **(E)**.

**Figure 6:**
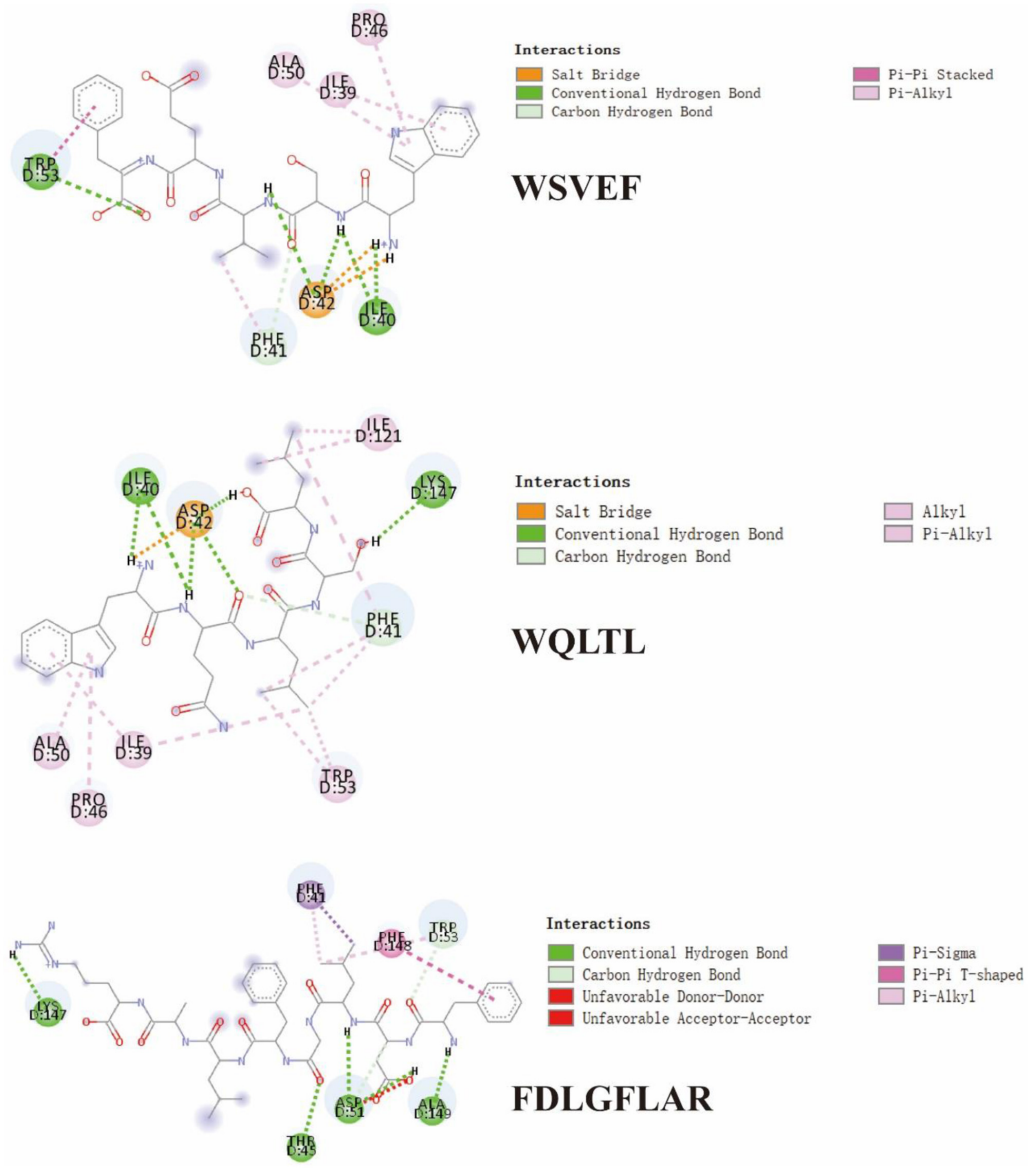
2D diagram showing interactions between TYIPs and tyrosinase amino acid residue.

In recent years, as a computer simulation technology, molecular docking has been widely used to study the possible interaction mechanism between inhibitors and enzymes (42–44). In order to show the binding mode of TYIPs with tyrosinase more intuitively, the AutoDock tool was used, and the results are shown in Figures 5, 6. Peptide DLGFLARGF bonded to the D chain of tyrosinase and interacted with its surrounding amino acid residues, nine hydrogen bonds were formed with Glu 340, Arg 111, Gly 115, Arg 108, Ile 96, Ser 95, Gly 113, Lys 5 and Glu 97 on the enzyme D chain (Figure 5C). In addition, amino acid residues Try 62, Pro 338, Pro 349, Glu 112, and Arg 111 can form a hydrophobic pocket on the enzyme D chain, which tightly surround and hold the peptide DLGFLARGF through hydrophobic action (Figure 5D). At the same time, electrostatic interaction also occurred between Glu 97, Glu 340, Glu 67, Asp 60 and the peptide DLGFLARGF on the D-chain (as shown in Figure 5E). These results intuitively indicated that hydrogen bonding was the main driving force (as shown in Table 4) involved in the interaction between peptide DLGFLARGF and tyrosinase. It can be inferred that the peptide DLGFLARGF binds to the inactive center of tyrosinase through hydrogen bonding, and indirectly inhibits the binding of substrate to tyrosinase by changing the conformation of the enzyme, thereby inhibiting the catalytic activity of the enzyme.

**Table 4:** Docking results according to hydrophobic interaction, electrostatic interaction and hydrogen bond for complex.

	**DLGFLARGF**	**WQLTL**	**WSEVF**	**FDLGFLAR**
Hydrogen bonds	Glu 340, Arg 111, Gly 115, Arg 108, Ile 96, Ser 95, Gly 113, Lys 5, Glu 97	Asp 42, Lys 147, Ile 40	Asp 42, Ile 40, Trp 53	Ala 149, Asp 51, Thr 45, Lys 147
Electrostatic interaction	Glu 97, Glu 340, Glu 67, Asp 60	Asp 42	Asp 42	No
Hydrophobic interaction	Tyr 62, Pro 338, Pro 349	Ile 121, Ile 39, Pro 46, Ala 50, Trp 53, Phe 41	Trp 53, Phe 41, Pro 46, Ala 50, Ile 39	Phe 148, Trp 53, Phe 41

In Figure 6 and Table 4, it could be seen that driving forces in the peptides WQLTL, WSEVF, and FDLGFLAR were less than DLGFLARGF, which also led to lower tyrosinase inhibitory activity of these peptides. Moreover, molecular docking can also be used to screen enzyme inhibitors based on binding energy changes (45, 46). The three peptides have different binding energies with WQLTL = −8.3 kcal/mol, WSEVF = −9.2 kcal/mol, FDLGFLAR = −6.4 kcal/mol, which was not completely consistent with the trend of tyrosinase inhibition ability in Figure 4. This may be because the most stable conformation was selected based on the lowest binding energy, but the peptide conformation in the reaction system may not be the most stable (47). On the other hand, it may also be due to the longer-chain of FDLGFLAR closer to the center of the hydrophobic cavity and the more hydrophobic interactions of WQLTL than WSEVF.

### The Effect of DLGFLARGF on B16F10 Melanoma Cells

**Figure 7:**
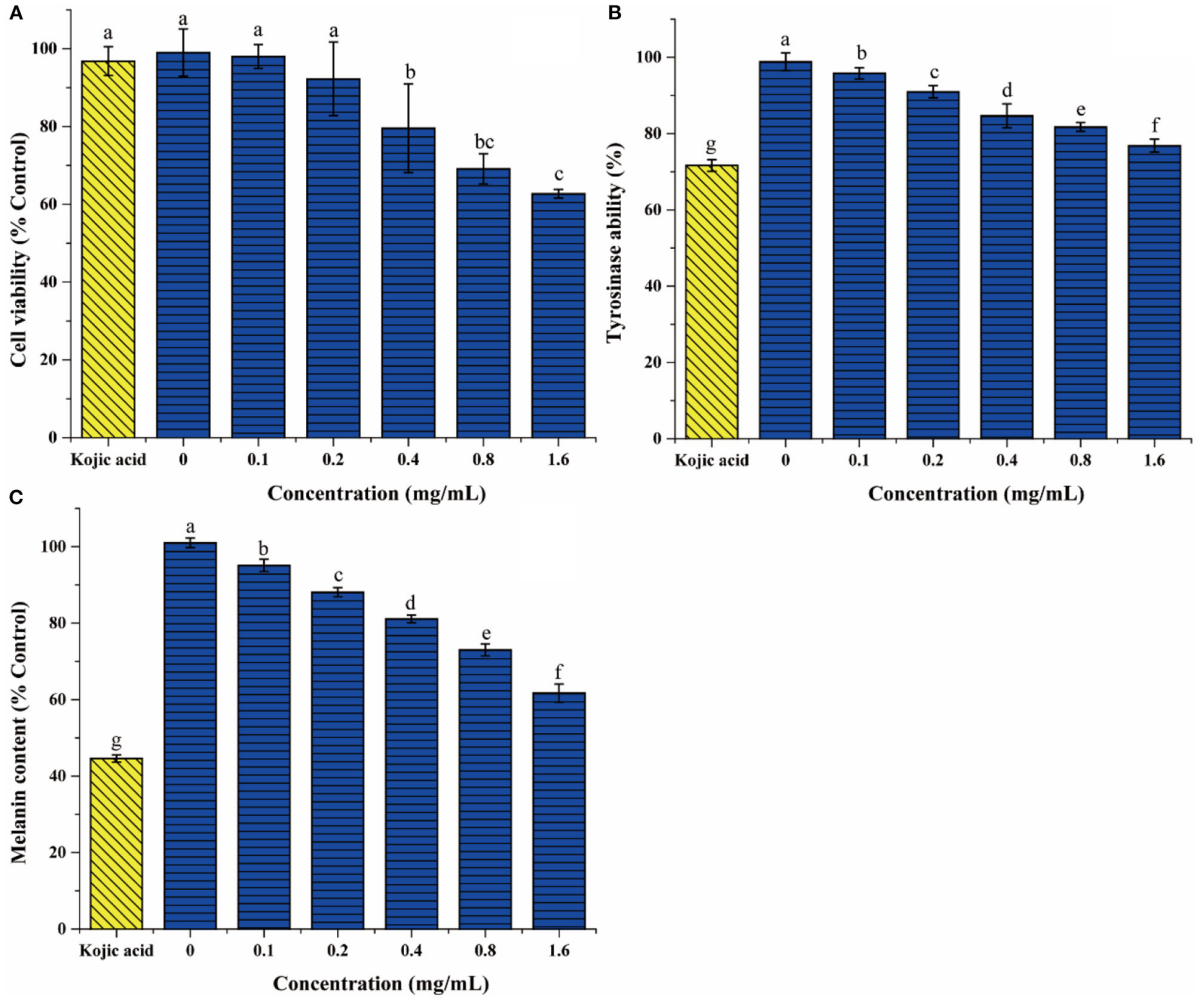
The effect of DLGFLARGF on B16F10 melanoma cells cytotoxicity **(A)**, tyrosinase activity **(B)**, and melanin content **(C)**. Kojic acid (0.75 mg/ml) was used as a positive control. The results are expressed as a percentage of the control, and the value is the mean ± SD of three independent experiments. According to Duncan's test, different letters indicate that the statistical difference between the two groups is *p* < 0.05.

The CCK-8 method was used to determine the potential cytotoxicity of different concentrations of DLGFLARGF compared with kojic acid (0.75 mg/ml). Figure 7A shows the effect of DLGFLARGF and kojic acid on the viability of B16F10 cells. With the increase of DLGFLARGF concentration, the cell survival rate gradually decreased. But within the tested concentration range (0–1.6 mg/ml), no obvious cytotoxicity was obtained (viability > 50%). Therefore, the concentrations of 0, 0.1, 0.2, 0.4, 0.8, and 1.6 mg/ml were selected to study the inhibitory effect of DLGFLARGF on melanin synthesis.

To further evaluate the tyrosinase inhibition of DLGFLARGF, B16F10 melanoma cells were treated with different concentrations of DLGFLARGF and kojic acid (0.75 mg/ml). The result is shown in Figure 7B. With the increase of DLGFLARGF concentration, tyrosinase activity gradually decreased, indicating gradually increased tyrosinase inhibitory ability. The inhibition rate was 23.19% for 1.6 mg/ml of DLGFLARGF, which was very close to that of kojic acid with 28.34% inhibition rate under 0.75 mg/ml. According to the previous results, the peptide DLGFLARGF was found to reduce tyrosinase activity by binding to the amino acid residue sites on the D chain of tyrosinase upon entry into the cells, but kojic acid in published study was proved to be docked to the catalytic site of mushroom tyrosinase and thus had a stronger tyrosinase inhibitory activity (48).

The content of melanin can directly determines the degree of skin whiteness. At present, the recognized process of melanin formation is roughly that tyrosine is oxidized to dopa under the action of tyrosinase, and then dopa is oxidized to dopa-quinone, which finally forms eumelanin through a series of reactions. Tyrosinase is the key enzyme for melanin formation (49). As shown in Figure 7C, the melanin content gradually decreased as the concentration of DLGFLARGF increased and showed a concentration-dependent relationship, indicating that DLGFLARGF can effectively inhibit the production of melanin. In terms of the inhibitory effects on melanin production, the melanin content was reduced by 55.4% in the cells treated with kojic acid (0.75 mg/ml), while DLGFLARGF reduced by 38.3% at 1.6 mg/ml. Kojic acid had been proved to be better than DLGFLARGF in inhibiting tyrosinase activity. On the other hand, the synthesis of melanin involves various factors besides tyrosinase, and kojic acid could also regulate transcription of tyrosinase pathway genes and bleach produced melanin (50). Moreover, collagen peptide has mild functional activity, easy absorption and high skin compatibility, while kojic acid lacks good permeability in application, and show high toxicity and low activity stability (51).

## Conclusions

In this work, the alcalase hydrolysate of grass carp fish scale gelatin was discovered to have promising tyrosinase inhibitory activity. The tyrosinase inhibition rate of fish scale gelatin treated with alcalase was 61.7% (at 5 mg/ml). The MW distribution of the hydrolysate was mainly below 3,358 Da (73.33%), and the content of amino acids that inhibit melanin was up to 35.44%. A rapid screening method of TYIPs based on bioaffinity ultrafiltration combined with LC-Orbitrap-MS/MS was established. 52 peptides were identified from PAT, among which 4 new peptides were screened. The peptide DLGFLARGF showed excellent tyrosinase inhibitory activity with an IC_50_ value of 3.09 mM. Hydrogen bonds were the predominant driving force in the interaction between peptide DLGFLARGF and tyrosinase according to molecular docking. In addition, when the concentration of DLGFLARGF reached 1.6 mg/ml, the melanin content and tyrosinase activity decreased to 61.7% and 76.81% of the control group, respectively. The above results indicate that bioaffinity ultrafiltration combined with LC-Orbitrap-MS/MS is an effective method for high-throughput screening of TYIPs. Moreover, DLGFLARGF can be widely used as a tyrosinase inhibitor in the whitening foods and pharmaceuticals.

## Data Availability Statement

The original contributions presented in the study are included in the article/supplementary material, further inquiries can be directed to the corresponding authors.

## Author Contributions

Z-ZH: experiments, data curation, and writing-original draft. X-MS: supervision, review, and editing. LZ: investigation, methodology, review, and editing. M-JZ: experiments. Z-CT: project administration, supervision, and funding acquisition. All authors contributed to the article and approved the submitted version.

## Funding

This study was supported by the National Key R&D Program of China (No. 2018YFD0901101).

## Conflict of Interest

The authors declare that the research was conducted in the absence of any commercial or financial relationships that could be construed as a potential conflict of interest. The reviewer LZ declared a shared affiliation with the author Z-CT to the handling editor at the time of review.

## Publisher's Note

All claims expressed in this article are solely those of the authors and do not necessarily represent those of their affiliated organizations, or those of the publisher, the editors and the reviewers. Any product that may be evaluated in this article, or claim that may be made by its manufacturer, is not guaranteed or endorsed by the publisher.
